# Combination of giant fibroadenoma and galactocele: two rare benign breast lesions in a 27-year-old mother presenting with a long-standing breast mass: a case report

**DOI:** 10.1097/RC9.0000000000000365

**Published:** 2026-03-10

**Authors:** Wondwosen Mengist Dereje, Ephrem Awoke Shiferaw, Asratu Getnet Amare, Rediet Tadesse Amare, Misgana Lemma Gurmu, Cheru Lilay Gebrehiwet

**Affiliations:** a Department of Neurology, College of Medicine and Health Sciences, University of Gondar, Gondar, Ethiopia; b Department of Pathology, College of Medicine and Health Sciences, University of Gondar, Gondar, Ethiopia; c Department of Surgery, College of Medicine and Health Sciences, University of Gondar, Gondar, Ethiopia

**Keywords:** benign tumor, case report, galactocele, giant fibroadenoma, surgical excision

## Abstract

**Introduction and Importance:**

A galactocele is a rare, benign cystic breast lesion caused by obstruction of the lactiferous ducts, leading to milk accumulation. Fibroadenoma is the most common benign breast tumor; however, giant fibroadenomas account for only 0.5% of cases. While isolated giant galactoceles and giant fibroadenomas have been reported as causes of long-standing breast masses, their simultaneous occurrence within a single mass has not been previously described. This case highlights the rare coexistence of these lesions and underscores the need for a multidisciplinary approach involving surgeons, pathologists, radiologists, and reconstructive specialists.

**Clinical Presentation:**

A 27-year-old para I woman presented with a left breast swelling of three years’ duration. It began as a small, palpable lump that gradually increased in size. She did not seek medical attention during the first 2 years. Over the past year, the mass enlarged rapidly, causing pain and pressure that disrupted her sleep and daily activities. Physical examination revealed a 20 × 15 cm mass in the left breast. Breast ultrasonography and fine-needle aspiration cytology were performed, and the patient opted for mastectomy. Histopathologic examination confirmed the diagnosis.

**Clinical Discussion:**

The coexistence of a giant fibroadenoma and a galactocele within a single breast mass is extremely rare. Radiologic imaging can support the diagnosis; however, definitive diagnosis often requires histopathologic evaluation.

**Conclusion:**

Although both giant fibroadenomas and galactoceles are benign lesions, they can mimic malignant breast tumors. Careful evaluation is necessary to distinguish them from malignancy and to ensure the early detection or exclusion of breast cancer.

## Background

A galactocele is an uncommon, benign cystic lesion of the breast that develops as a result of obstruction within the lactiferous ducts^[^1,3^]^. This blockage leads to the accumulation of milk within the duct, forming a cystic mass. Although galactoceles can occur in women of any age, they are most frequently observed in postpartum women, particularly during or after the lactation period^[^4,5^]^. The lesion is typically slow-growing, well-circumscribed, and often painless, although it may occasionally cause discomfort or local swelling depending on its size and location within the breast.

Fibroadenomas are common breast lesions that typically occur in women under 30 years of age. They represent a polyclonal proliferation of both epithelial and stromal tissues and are considered hyperplastic lesions rather than true neoplasms. Clinically, fibroadenomas usually present as firm, mobile, painless nodules with well-defined margins and are easily palpable^[^6^]^.

This case is reported in accordance with the SCARE guidelines^[^7^]^.

## Clinical presentation

A 27-year-old para I woman presented with a left breast swelling of 3 years’ duration. The swelling began as a small, palpable mass in the upper outer quadrant of the left breast while she was breastfeeding her daughter. At that time, she was not concerned because the mass was small, and friends had reassured her that it would subside with continued breastfeeding. Despite exclusive breastfeeding for the first 6 months and continued breastfeeding until her daughter was 2 years old, the swelling persisted.

Over the past year, the swelling increased rapidly in size and was accompanied by pain and a pressure sensation that interfered with her sleep and routine daily activities. She had been using an implant contraceptive method for the past 2 years. Her menstrual cycles were regular, occurring every 25–30 days and lasting 3–5 days.

She denied symptoms such as headache, visual disturbances, or cranial nerve palsies.

On presentation to our clinic, the patient appeared comfortable. Her vital signs were stable: blood pressure 100/65 mmHg, pulse rate 82 beats per minute, respiratory rate 18 breaths per minute, temperature 36.7 °C, and oxygen saturation 98% on room air.

**Figure 1.**
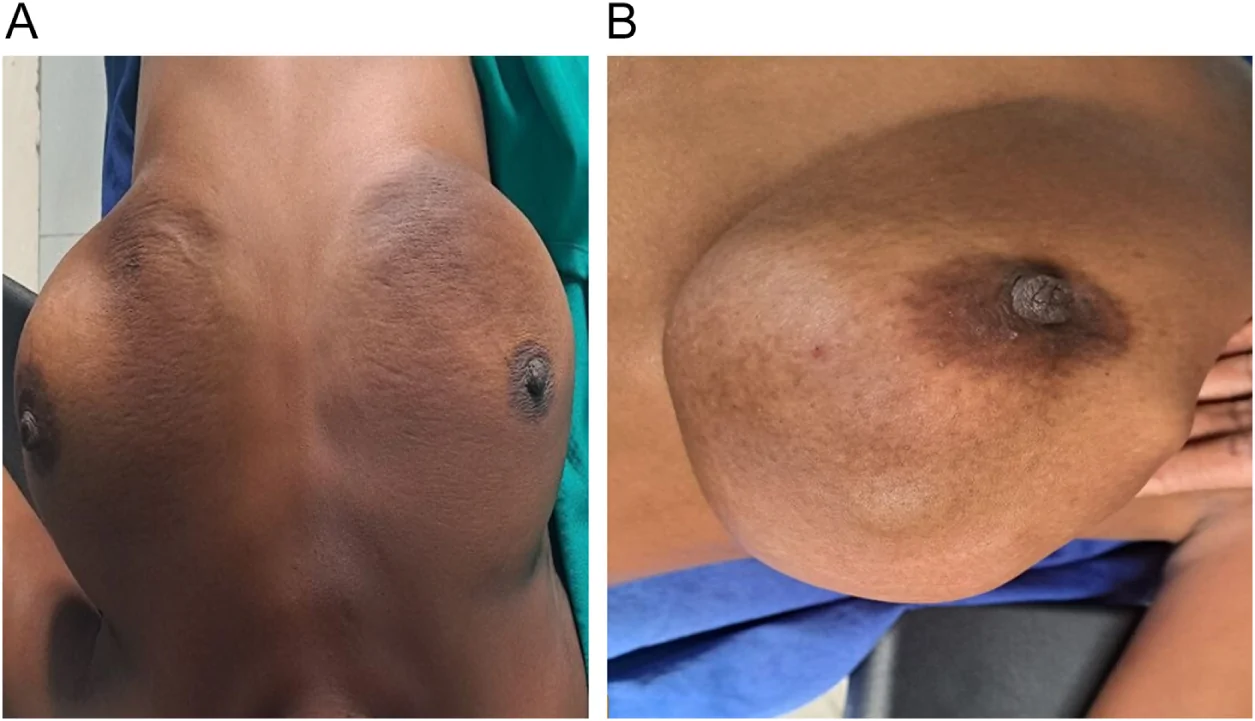
Enlarged left breast compared to right breast (1A) and visible breast mass (1B).

Breast examination revealed a large, non-tender, palpable mass measuring approximately 20 × 15 cm, occupying the upper and lower outer quadrants of the left breast (Fig. 1A,1B). No axillary lymphadenopathy was detected, and examination of the contralateral breast was unremarkable.

Given the rapid increase in size over the past year, a phyllodes tumor was considered a possible diagnosis. In view of her reproductive age, a giant fibroadenoma was also included in the differential diagnosis.

Urine human chorionic gonadotropin (hCG): negative.

**Figure 2.**
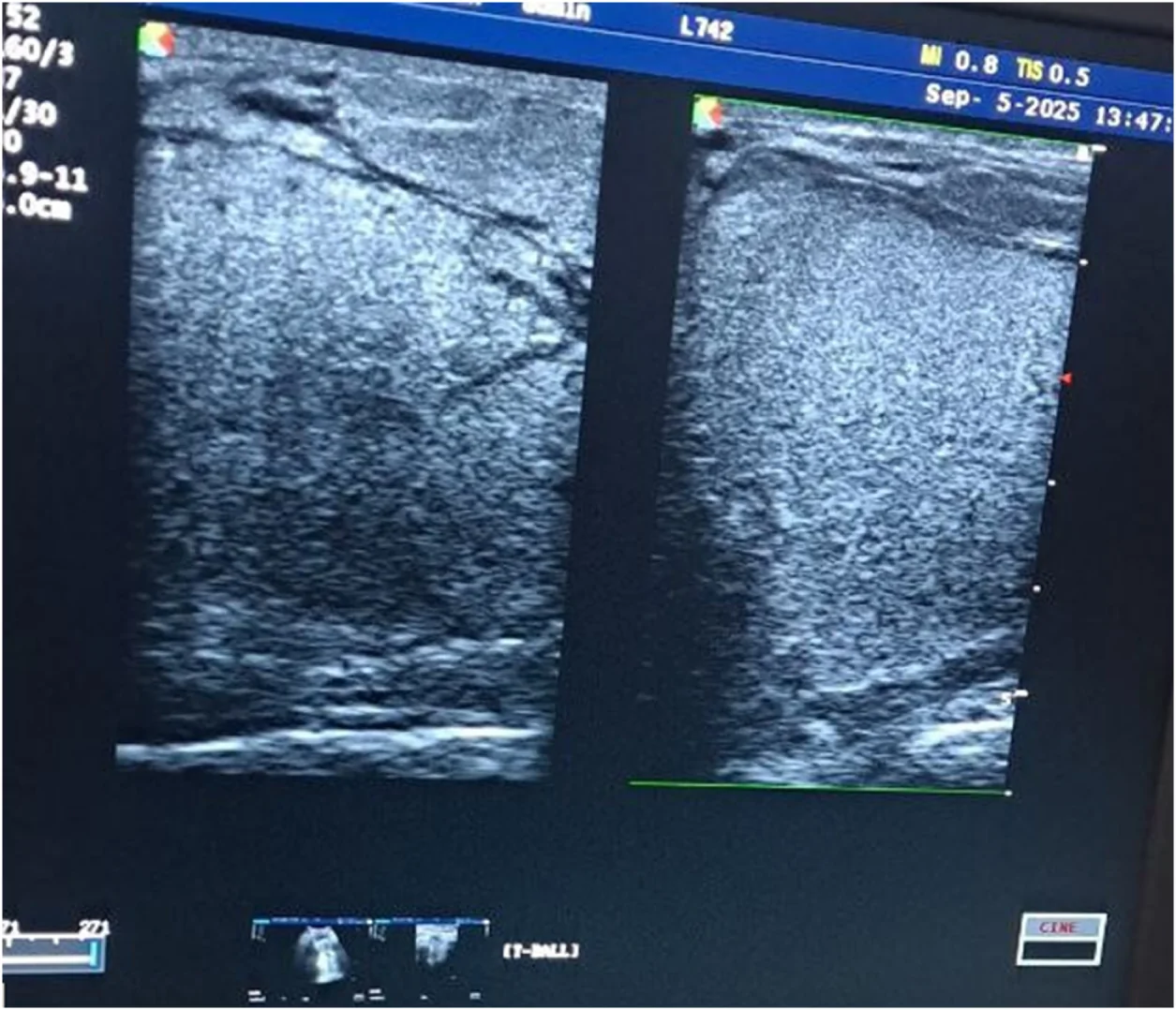
Ultrasound image showing a large, loculated, hypoechoic cystic lesion with regular margins, suggestive of a galactocele.

Breast ultrasonography demonstrated a large, multiloculated, hypoechoic cystic lesion with regular margins and posterior acoustic enhancement, suggestive of a galactocele (Fig. 2). Based on these findings, a galactocele was added to the differential diagnosis. Fine-needle aspiration cytology (FNAC) was performed and revealed features consistent with a long-standing galactocele.

**Figure 3.**
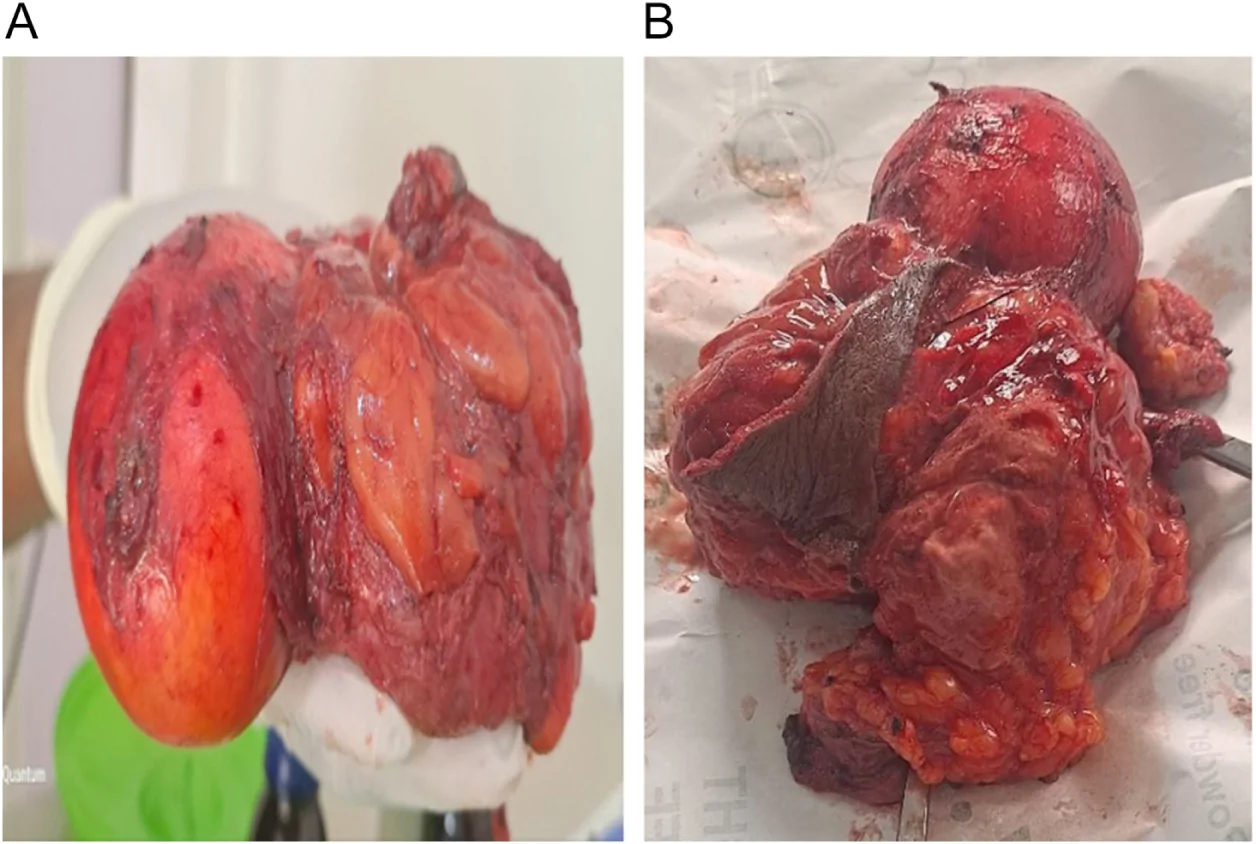
3A–3B: The image of the excised mass from different angle.

**Figure 4.**
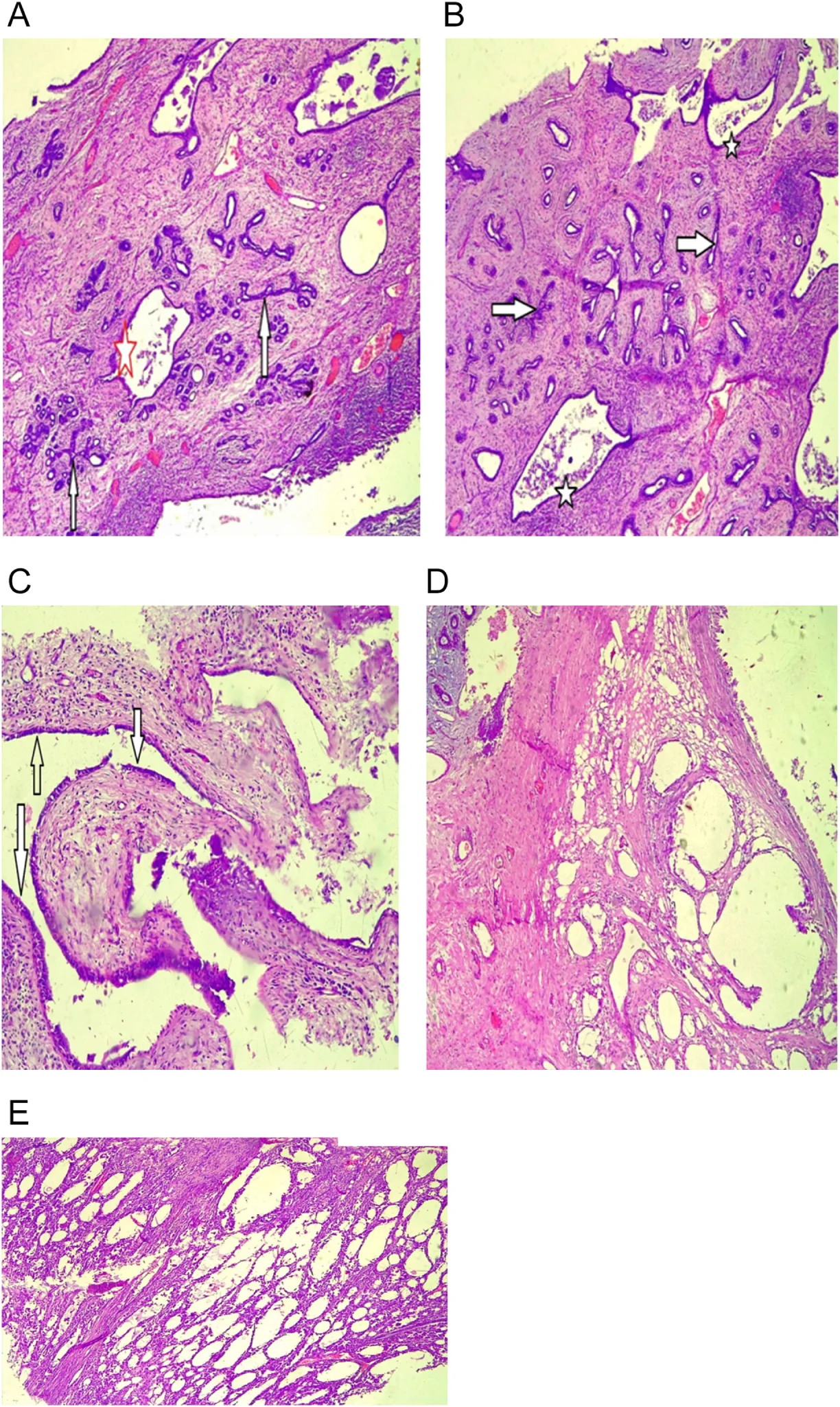
(A) Image showing glandular proliferation with predominantly having intracanalicular growth pattern (arrow) and dilated glands (star) (Features of Fibroadenoma). (B) Image showing glandular proliferation with predominantly having intracanalicular growth pattern (arrow) with few dilated glands containing secretory material (star) within surrounding stroma composed of bland looking oval to spindle cells (Features of Fibroadenoma). (E) Image showing breast tissue with lactational changes. (A–E) Histopathologic images with both features of galactocele and fibroadenoma.

After discussion of the findings with the patient, she opted for surgical excision because of the significant discomfort caused by the mass. Following the provision of informed written consent, she was taken to the operating room. The patient was positioned supine and placed under general anesthesia. The operative field was properly draped, and antisepsis was performed using an iodine solution. A skin incision was made, followed by careful dissection of the underlying soft tissues. The mass was gently separated from the surrounding tissues and completely excised (Fig. 3A,3B). Hemostasis was secured, and the nipple–areolar complex was preserved. The excised specimen was sent for histopathologic evaluation, which confirmed the presence of both a galactocele and a fibroadenoma (Fig. 4A–4E).

The patient was transferred to the post-anesthesia care unit, where she remained for 8 hours, and was then transferred to the recovery unit, where she stayed for 24 hours. She was subsequently transferred to the ward for follow-up.

HIGHLIGHTS

- Galactocele is a benign cystic breast lesion caused by obstruction of lactiferous ducts, commonly occurring during lactation, and may mimic other breast masses, necessitating careful evaluation to avoid misdiagnosis.
- Giant fibroadenomas are rare benign breast tumors, often disfiguring due to their size, but carry no malignant potential and most frequently occur in young or lactating women.
- Ultrasound-guided fine-needle aspiration provides both diagnostic confirmation and therapeutic relief, though surgical excision is required for large, long-standing, or firm lesions.
- Management of these lesions prioritizes complete excision while preserving breast aesthetics, with reconstructive surgery rarely needed, highlighting the importance of careful planning for cosmetic outcomes.
- This case highlights the exceptionally rare combination of two benign breast lesions and underscores the importance of a multidisciplinary approach.

The patient had an uneventful postoperative recovery and was discharged after 3 days with an outpatient follow-up appointment. At her 3-week postoperative visit, the surgical wound was well healed, and she reported no new complaints. She was subsequently discharged from follow-up.

## Discussion

A galactocele, also referred to as a lactocele, is a cystic lesion of the breast that contains milk or a milky fluid. It arises from obstruction of a lactiferous duct, leading to the accumulation of secretory fluid within the duct. The majority of cases occur during pregnancy, particularly in the lactational period^[^1^]^. The frequency of galactoceles varies depending on the patient’s age and pregnancy status. For instance, among pregnant women evaluated for breast-related complaints such as pain, palpable nodules, or unilateral spontaneous failure of lactation, the reported prevalence of galactoceles ranges from 20%^[^6^]^ to 44%^[^8^]^. These variations highlight the importance of considering patient demographics and reproductive status when assessing cystic breast lesions.

Giant fibroadenomas, defined as fibroadenomas measuring more than 5 cm in diameter or weighing over 500 g, are rare benign breast lesions that account for approximately 0.5%–2% of all fibroadenomas. They most commonly occur in adolescent females or in women during pregnancy or lactation^[^9^]^.

Evidence from endocrinology and histopathologic studies, including both macroscopic and microscopic examinations, suggests that the pathogenesis of galactocele involves a combination of factors: current or prior stimulation by prolactin, secretory activity of the breast epithelium, and obstruction of the lactiferous ducts, leading to their dilation with accumulated milk from the surrounding breast tissue^[^10,11^]^. Clinically, galactoceles may present as a single mass or multiple masses, particularly in lactating women. They can occur unilaterally or bilaterally and are typically non-tender, firm, well-circumscribed, discrete, and freely movable. In some cases, the lesion may be associated with milky nipple discharge; however, this finding is not consistently observed^[^10–14^]^.

Fibroadenomas typically present as rubbery, mobile, and non-tender breast masses. A giant fibroadenoma, however, can be disfiguring and may impede the growth of normal breast tissue due to its direct pressure effect, although it carries no malignant potential.

In certain instances, a galactocele can mimic other neoplastic or non-neoplastic breast masses, potentially leading to diagnostic confusion among clinicians, particularly when advanced imaging or histopathologic facilities are not readily available^[^2,5,10,12^]^.

Galactoceles can resemble several breast pathologies, including cysts, fibroadenomas, phyllodes tumors, abscesses, and malignancies^[^12^]^. Accurate differentiation is essential to prevent unnecessary interventions and ensure appropriate management.

The differential diagnosis of fibroadenomas includes galactocele, breast abscess, and breast cancer. In cases of giant fibroadenoma, the primary differential diagnoses are phyllodes tumors and pseudoangiomatous stromal hyperplasia^[^10^]^.

Differentiating fibroadenomas from phyllodes tumors is clinically important because of differences in therapeutic strategies. Fibroadenomas can be safely monitored or simply enucleated, whereas phyllodes tumors require surgical excision^[^15^]^. Furthermore, phyllodes tumors must be resected with adequate margins – most commonly recommended as 1 cm – to minimize the risk of local recurrence^[^7,16^]^.

Ultrasound-guided fine-needle aspiration (FNA) serves a dual purpose in most cases, providing both a definitive diagnosis and therapeutic relief by decompressing the cystic lesion. However, in situations where the galactocele is markedly enlarged, firm, or contains solid components, surgical excision may be necessary to completely remove the mass and alleviate associated symptoms^[^3^]^.

Although a phyllodes tumor was initially considered, the ultrasound examination revealed a well-circumscribed, cystic lesion with regular margins, suggestive of a galactocele. Despite experiencing pain and discomfort from the breast swelling, the patient denied weight loss, night sweats, or loss of appetite, reducing the likelihood of breast cancer. Furthermore, the ultrasound findings and FNAC results demonstrated thick, milky fluid containing lipid-laden macrophages, supporting the diagnosis of galactocele.

The most common sonographic appearance of fibroadenomas in pregnant and lactating women is similar to that in non-gestational patients: an oval or round mass with a wider-than-tall orientation, circumscribed margins, and occasional gentle lobulations^[^4^]^. On mammography, fibroadenomas typically appear well-circumscribed, round or oval in shape, and may be smoothly lobulated^[^17^]^. Coarse, popcorn-like calcifications can be observed if the tumor has undergone infarction; otherwise, calcifications are uncommon, as these lesions generally occur in younger patients^[^17^]^.

The sonographic appearance of galactoceles is variable and depends on the fat content and the age of the lesion. Galactoceles are typically round or oval and may appear anechoic, hypoechoic, or echogenic. In cases of chronic inflammation, wall thickening may be observed^[^10^]^. As the lesion ages, internal echogenicity usually increases, and a fat–fluid level may occasionally be seen^[^8^]^. Even when internal complex echoes are present, vascular flow should not be observed; however, hyperemia may be noted in the adjacent compressed breast parenchyma^[^8^]^.

On mammography, the presence of a mass exhibiting a fat–fluid level on the lateral projection is a diagnostic feature of galactocele in the appropriate clinical context^[^10^]^. Advanced imaging, such as MRI or CT, is indicated when breast cancer is suspected or when assessment of the extent of local and regional disease is required.

Although imaging is not definitive for diagnosing breast lesions, it plays a crucial role in narrowing the differential diagnosis and guiding management strategies.

Malignant transformation of a fibroadenoma occurs in less than 0.3% of cases^[^17^]^. In contrast, the long-term prognosis of galactocele appears to be favorable. For example, five patients who developed galactocele during pregnancy were followed for 27 months, and none developed breast carcinoma. However, the authors of this manuscript recommend further studies to evaluate the potential correlation between galactocele and breast cancer, as the available data are limited by short follow-up duration and small sample size.

The coexistence of benign and malignant breast lesions has been described in the literature. Although no exact percentage quantifying this correlation has been established, several case reports have documented such associations. Borecky N *et al* reported a case of coexisting fibroadenoma and breast cancer^[^18^]^.

Published studies indicate that the predominant type of carcinoma arising within a fibroadenoma is noninvasive or in situ carcinoma (80–95%), most commonly lobular carcinoma in situ^[^19,20^]^.

Tayae M et al. described the coexistence of a giant galactocele and prolactinoma^[^21^]^; however, despite a thorough literature review, no cases of coexisting galactocele and breast cancer have been identified. Based on the available evidence, we believe that the risk of malignant transformation in galactocele, if any, is very low.

A thorough literature review revealed only one published case describing the coexistence of galactocele and fibroadenoma. Vuolo M *et al* reported a case of concurrent galactocele and fibroadenoma^[^22^]^. Even in this previously reported case, neither the galactocele nor the fibroadenoma was classified as giant.

In our report, both pathological entities were giant. To the best of our knowledge, this represents the first reported case describing the coexistence of a giant fibroadenoma and a giant galactocele.

Tissue biopsy is essential for diagnosing large breast lesions. FNA biopsy is limited in its ability to distinguish phyllodes tumors from giant fibroadenomas, whereas core needle biopsy provides greater diagnostic accuracy^[^23^]^.

In the reported case, a core needle biopsy was not performed, as the clinical presentation, FNAC findings, and ultrasound images were all suggestive of a galactocele. Surgical excision was undertaken primarily for management, as the patient opted for this procedure and the histopathologic evaluation confirmed the diagnosis.

This case also underscores that aspiration may not be a feasible treatment option for long-standing or large galactoceles. In such situations, surgical excision becomes necessary to completely remove the breast mass. Careful consideration of postoperative cosmesis is essential, particularly because most patients affected by galactoceles are young and concerned about the aesthetic outcome of the operated breast.

Additionally, the primary goal of treatment for giant fibroadenoma is complete excision of the tumor while preserving the nipple–areolar complex and achieving breast symmetry. It was previously thought that reconstructive procedures were usually required after the removal of large breast lesions to restore bilateral symmetry^[^23^]^. However, a recent systematic review demonstrated that reconstructive surgery is rarely necessary in cases of giant fibroadenoma^[^24^]^.

## Conclusion

Both giant fibroadenoma and galactocele are benign cystic breast lesions. Galactocele arise due to lactiferous duct obstruction, most commonly occurring during lactation. Although usually straightforward to diagnose, they can occasionally mimic neoplastic or other benign breast masses, requiring careful evaluation to avoid misdiagnosis and unnecessary interventions. Management typically involves ultrasound-guided aspiration, but surgical excision may be necessary for large, long-standing, or complex lesions, with attention to cosmetic outcomes in affected patients.

## Patient perspective

I had experienced the swelling for 3 years. Initially, I was not concerned, as I hoped it would resolve on its own; however, instead of decreasing, it continued to enlarge, which became increasingly frightening. Over the past year, my breast size increased significantly, and the associated pain became quite bothersome. The mass and its pressure effects had a substantial impact on my social life I was no longer able to wear my favorite outfits. More than anything, I was deeply worried that it might be breast cancer or a condition progressing toward malignancy.

Following surgery, I have been able to resume my social life as it was before the illness. Learning that the pathological results showed no evidence of cancer was the best news I could have received. Cosmetically, as the wound has healed, my breast feels normal, with only a small scar remaining.

## Data Availability

The data that support the findings of this study are available from the corresponding author upon reasonable request.

## Methods

The study has been reported in line with SCARE criteria.
